# Hypoxia-Induced MicroRNA-210 Targets Neurodegenerative Pathways

**DOI:** 10.3390/ncrna4020010

**Published:** 2018-03-27

**Authors:** Michelle E. Watts, Sarah M. Williams, Jess Nithianantharajah, Charles Claudianos

**Affiliations:** 1 Queensland Brain Institute, The University of Queensland, Brisbane QLD 4072, Australia; m.watts1@uq.edu.au; 2 Monash Bioinformatics Platform, Monash University, Melbourne VIC 3800, Australia; sarah.williams1@monash.edu; 3 The Florey Institute of Neuroscience & Mental Health, The University of Melbourne, Melbourne VIC 3052, Australia; jess.n@florey.edu.au; 4 Centre for Mental Health Research, The Australian National University, Canberra ACT 2601, Australia

**Keywords:** microRNA, hsa-miR-210, microRNA targeting, SH-SY5Y cells, neurodegeneration

## Abstract

Hypoxia-regulated microRNA-210 (miR-210) is a highly conserved microRNA, known to regulate various processes under hypoxic conditions. Previously we found that miR-210 is also involved in honeybee learning and memory, raising the questions of how neural activity may induce hypoxia-regulated genes and how miR-210 may regulate plasticity in more complex mammalian systems. Using a pull-down approach, we identified 620 unique target genes of miR-210 in humans, among which there was a significant enrichment of age-related neurodegenerative pathways, including Huntington’s, Alzheimer’s, and Parkinson’s diseases. We have also validated that miR-210 directly regulates various identified target genes of interest involved with neuronal plasticity, neurodegenerative diseases, and miR-210-associated cancers. This data suggests a potentially novel mechanism for how metabolic changes may couple plasticity to neuronal activity through hypoxia-regulated genes such as miR-210.

## 1. Introduction

In recent years, increasing evidence has established microRNAs (miRNA) as critical regulators of neuronal development and plasticity, with new roles for neuronal miRNAs continually emerging. A critical aspect of complex plasticity processes is dynamic gene regulatory mechanisms that can act locally and promote appropriate changes within discrete synapses in response to stimuli [1]. Single miRNAs are capable of regulating up to hundreds of different target genes, primarily through imperfect base pairing within 3′ untranslated regions (3′-UTRs) of target messenger RNAs (mRNAs) [2]. miRNAs have evolved as highly configurable regulatory elements which themselves are subject to complex temporal and spatial regulation through controlled transcription, biogenesis, and degradation. Within the brain, rapid turnover of miRNAs has been identified by various activity-dependent mechanisms, including activation of the miRNA processing enzyme Dicer, calpain-mediated cleavage from the post-synaptic density (PSD), and activity-induced local degradation of RNA-induced silencing complex (RISC) proteins [3]. There is also an enrichment of miRNA expression in the brain, and various individual miRNAs have already been directly implicated in regulating plasticity [4,5].

Recently, we examined changes in gene expression following long-term memory formation using olfactory conditioning in the honeybee [6]. This identified a broad downregulation of protein-coding genes and a corresponding upregulation of noncoding RNA genes. Among validated noncoding genes, miRNA-210 (miR-210) exhibited the highest upregulation following olfactory conditioning and was expressed in relevant learning-related regions of the honeybee brain. Knockdown of miR-210 in the honeybee also revealed impairments in long-term recall following olfactory conditioning, providing the first evidence that miR-210 may be involved in learning and memory.

Found across vertebrates and invertebrates, miR-210 is a highly conserved, hypoxia-regulated miRNA, modulated through a hypoxia response element (HRE) in its promoter [7]. In all tissue and cell types examined, miR-210 has been upregulated under hypoxic conditions, and it is considered to be the master miRNA regulator of hypoxia response involved in numerous functions, including mitochondrial respiration, DNA repair, cell proliferation, and angiogenesis [8,9]. There have been few studies of miR-210 in the nervous system so far, but ectopic miR-210 overexpression has been shown to stimulate angiogenesis and proliferation of both embryonic and adult neural progenitors within the mouse brain subventricular zone [10,11]. Various studies have also found miR-210 to be significantly upregulated in rodent models of epilepsy at multiple time points using differing induction methods, as well as in vitro, in an *N*-methyl-d-aspartate (NMDA) receptor dependent manner following exposure of primary rat neurons to soluble amyloid β, a pathogenic component of Alzheimer’s disease (AD) [12,13,14,15,16,17]. Given this data, there appears to be a strong correlation of miR-210 regulation with neuronal activation in rodent models and a potentially conserved role in learning and memory in mammalian systems.

This pertains to the broader hypothesis that oxygen use and physiological induction of hypoxia may act as an activity-dependent modulator of gene expression in the brain and whether hypoxia-regulated genes may in turn influence plasticity. Oxygen metabolism is especially critical to the brain, being the largest source of energy consumption and having highly dynamic metabolic requirements. This heterogeneity in metabolic activity is driven by neuronal activity, and it is likely that mechanisms involved in metabolic regulation play a major role in directing plasticity; however, there has been limited research in this area. Here we used a global transcriptome pull-down approach to experimentally identify human mRNA targets of miR-210. We also identify novel miR-210-associated pathways by gene ontology analysis and validate that miR-210 directly regulates key genes associated with plasticity and neurodegeneration. Our data supports a potential role for hypoxia-induced miR-210 in the regulation of neurodegenerative pathways that may be relevant to both neurological disorders and normal cognitive function.

## 2. Results

### 2.1. Biotin Pull-Down of miRNA-210 Targets

**Figure 1:**
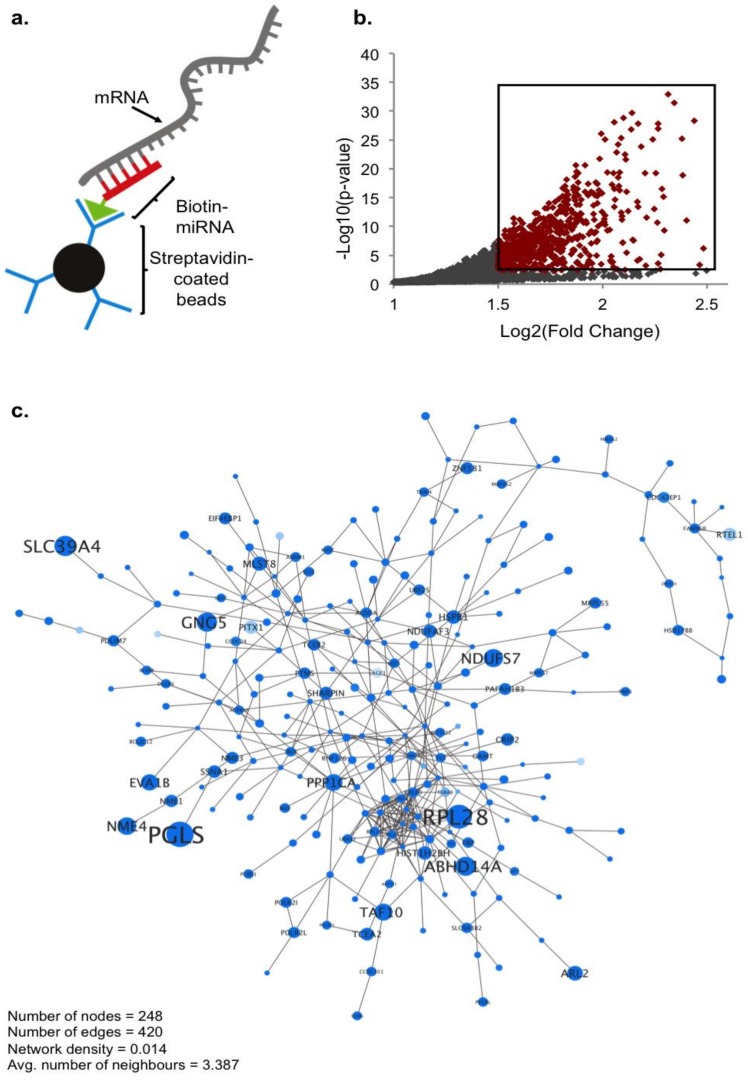
RNA sequencing (RNAseq) identification of genes enriched by miR-210 pull-down. (**a**) Schematic of the biotin–streptavidin pull-down approach used to isolate miR-210 target RNA. (**b**) Scatter plot of pull-down RNAseq data: 676 genes, highlighted in red, were found to be significantly enriched by hsa-miR-210 compared to cel-miR-239b using the following thresholds: Log2 (fold change) > 1.5, adjusted *p*-value < 0.05, *n* = 3 biological replicates, DEseq2: Wald “greater” test of significance, log fold change (lfc) threshold = 1. (**c**) Interactome network of genes significantly enriched by miR-210. Increasing node and font size correlate to increasing fold change; decreasing node saturation correlates to increasing *p*-value.

While a selection of miR-210 targets have already been identified in different cell types, not much is known about specific genes miR-210 may target in the nervous system. As a computational prediction of bona fide miRNA targets is challenging due to many miRNA recognition elements (MREs) having noncanonical base pairings, we utilized an unbiased sequencing approach to form a global picture of miR-210 targeting in a neuronal system [18,19]. As a model system, we chose the human-derived neuroblastoma cell line SH-SY5Y, commonly used in neuronal studies. SH-SY5Y also seemed an appropriate model for detecting functional targets of miR-210, as it is a cell line where miR-210 is expressed and known to be modulated in response to brain-derived neurotrophic factor (BDNF) differentiation and 1-methyl-4-phenyl-1,2,3,6-tetrahydropyridine (MPTP) exposure [20,21]. Isolation of mRNA targets was performed using a biotin pull-down technique first described by Orom et al. and more recently modified to efficiently detect translationally regulated miRNA targets [22,23]. miRNA mimics were designed and synthesized as sense-strand miRNA oligo sequences using 2′ *O*-methyl modified RNA bases that are less sensitive to degradation. A biotin molecule was conjugated to the 3′ end of the oligos to minimize potential binding interference with the 5′ miRNA seed region. Cells were transfected with human miR-210 mimic (hsa-miR-210-3p) alongside the negative control *Caenorhabditis elegans* miRNA, miR-239b (*cel*-miR-239b-5p) reported to have no known homology in the human genome. Cells were incubated with biotin–conjugated mimics for 24 h before lysis and isolation of target RNA using streptavidin-coated beads (Figure 1a).

Captured RNA was sequenced in triplicate using the IIlumina Hi-Seq system, and read counts generated from HTSeq were analyzed using DESeq2. RNA targets were identified as genes with significantly greater enrichment in miR-210 compared to control miR-239b pull-downs. From this we identified a total of 676 genes as potential miR-210 targets, of which 620 were unique targets, previously unassociated with miR-210 (significance cutoff: adjusted *p*-value < 0.05, Log2 (fold change) > 1.5) (Table S1) (Figure 1b,c). The top 20 genes that displayed the most significant enrichment by miR-210 are listed in Table 1. Among these 20 targets are multiple genes with an already established role in neuronal/synaptic plasticity (highlighted rows, Table 1). Particular genes of interest include *GRINA* (padj = 1.02 × 10^−25^, FC = 4.16), a subunit of the NMDA-receptor complex, and the translation initiation binding protein *EIF4EBP1* (*4E-BP1*) (padj = 5.55 × 10^−23^, FC = 4.04), known to be phosphorylated by neuronal activation signaling pathways and thought to be involved in regulating local gene expression in an activity-dependent manner [24,25]. Transmembrane protein TMUB1 (padj = 3.78 × 10^−23^, FC = 4.25) is also involved in synaptic transmission by regulating surface expression of α-amino-3-hydroxy-5-methyl-4-isoxazolepropionic acid (AMPA) receptors containing GluR2 subunits, and the adaptin AP2 complex subunit *AP2S1* (padj = 1.46 × 10^−25^, FC = 4.50) modulates endocytosis of AMPA and gamma-aminobutyric acid (GABA_A_) receptors [26,27]. As an additional validation of the RNA sequencing (RNAseq) data, we confirmed the significant enrichment of a selection of target genes by real-time quantitative PCR (qPCR) using control lysate sample expression to normalize the data for relative quantification (Figure S1) (validated genes indicated in Table 1).

To further identify genes from this target list that might be significant to the role of miR-210 in learning and memory, we generated a list of homologous human genes from genes identified previously in our lab as differentially regulated in honeybee olfactory conditioning [6]. Additionally, we compiled data from other in vivo studies profiling gene expression during long-term consolidation with available published gene expression data (Table S2). This included rodent studies of both chemically induced long-term potentiation and behavioral long-term consolidation paradigms [28,29,30,31,32,33,34,35,36,37,38]. From this we identified 14.94% of miR-210 enriched genes as differentially regulated in at least one study of long-term consolidation. This included a number of pull-down target gene homologs inversely correlated with miR-210 upregulation during honeybee olfactory conditioning (*ACTB*, *GAPDH*, *HSPB1*, *REEP6,* and *TECR*) as well as targets differentially regulated across multiple long-term consolidation studies (*ACTB*, *ARID5A*, *C11orf96*, *C1QTNF1*, *CAPNS1*, *CRABP1*, *GAPDH*, *HSPB1*, *TIMP1*, and *VGF*).

### 2.2. Global Ontology Analysis of miR-210 Targeting

To gain further insight into the functionality of miR-210, we used the publicly available Database for Annotation, Visualization, and Integrated Discovery (DAVID) functional annotation tool to examine gene ontology (GO) enrichment of miR-210 targets identified by pull-down RNAseq [39]. The most significantly enriched biological processes and Kyoto Encyclopedia of Genes and Genomes (KEGG) pathways among miR-210 targets are summarized in Table 2 (*p* < 0.1). The full list of significantly enriched biological processes (Table S3) includes 138 GO terms. These terms are primarily associated with the broader functionalities of small molecule synthesis (26.8%), energy metabolism (20.3%), macromolecule synthesis (10.1%), transcription/RNA synthesis and processing (7.2%), and translation/protein synthesis (5.8%). In addition, a number of GO processes previously associated with miR-210 were enriched: cell proliferation (GO:0008284, GO:0001938, GO:000936), cellular response to stress (GO:0006976, GO:0033554), anti-apoptosis (GO:0006916), and DNA repair (GO:0006307).

KEGG pathway analysis of the target gene set was also consistent with known regulatory functions of miR-210, including oxidative phosphorylation (OXPHOS) (*p* = 0.0089) and the vascular endothelial growth factor (VEGF) signaling pathway (*p* = 0.0212) (Table 2 and Table S4) [10,40]. Enriched KEGG pathways also included multiple cancers previously associated with miR-210 either by its identified oncogenic role (bladder cancer, *p* = 0.070) or its elevated expression (renal cell carcinoma, *p* = 0.045, and acute myeloid leukemia, *p* = 0.064) [41,42]. Of interest, multiple neurodegenerative diseases were also represented among miR-210 target genes, including Huntington’s disease (*p* = 0.002) Alzheimer’s disease (*p* = 0.018), and Parkinson’s disease (*p* = 0.049). Also significantly enriched and relevant to neurodegeneration was the mammalian target of rapamycin (mTOR) signaling pathway (*p* = 0.044). In AD patients, there is a significant upregulation of phosphorylated mTOR at serine 2481 (p-mTOR(Ser2481)) and a significant correlation between p-mTOR(ser2481) and tau phosphorylation. Total levels of mTOR target 4E-BP1 are also increased in AD patients, and p-4E-BP1 is correlated with increased levels of total tau [43]. Dysregulation of miR-210 itself has also been correlated with AD being downregulated in brain samples within the anterior temporal cortex, frontal cortex, and hippocampal regions as well as in cerebrospinal fluid and serum of AD patients [44,45,46]. This data indicates that miR-210 may have a role in neurodegenerative disorders and supports a potentially significant neuronal function of miR-210.

Additionally, within this gene set we looked at overrepresented transcription factor binding sites (TFBSs) using the oPOSSUM single site analysis tool (significance cutoff: Z-score > 15, target rate to background ratio >1.5) (Table S5) [47]. miR-210 already has a well-established role in the regulation of hypoxia, being induced under hypoxic conditions through the hypoxia-inducible factor (HIF) TFBS in its promoter. Moreover, a number of other genes transcriptionally regulated through HIF have previously been identified as miR-210 regulatory targets, indicating that miR-210 may act, at least in part, as a downstream modulator of HIF. Among our target gene set, the heterodimeric HIF-1A–Arnt transcription factor complex was one of 30 overrepresented transcription factors, with a total of 410 target gene hits (60.65% of targets) (Z-score: 65.1, target/background ratio: 1.75). We also looked at KEGG pathway annotations for all enriched transcription factors, which highlighted functionalities similar to miR-210 (Figure S2). Overall, enriched functionalities in gene ontology analysis are consistent with already known roles of miR-210 and indicate that identified biotin–miR-210 pull-down targets may be functionally significant regulatory targets of miR-210.

### 2.3. Validation of miR-210 Target Regulation

While identification of mRNAs pulled down by biotin–miR-210 highlighted genes potentially binding miR-210, this may not necessarily indicate a functional interaction. To further confirm whether genes are direct regulatory targets of miR-210, various genes of interest were chosen for dual-luciferase assay validation. Selection of genes of interest was based on associated gene ontology and/or known neuronal function. We looked at genes that were annotated to multiple enriched KEGG pathways (Table S6), which highlighted a number of metabolic genes overlapping between OXPHOS and Huntington’s, Alzheimer’s, and Parkinson’s disease pathways. This consisted of genes encoding multiple subunits of the electron transport chain (ETC) complexes I, III, IV, and V (Figure S3). There was also significant overlap across miR-210-associated cancers and the VEGF and mitogen-activated protein kinase (MAPK) signaling pathways (Table S6).

**Figure 2:**
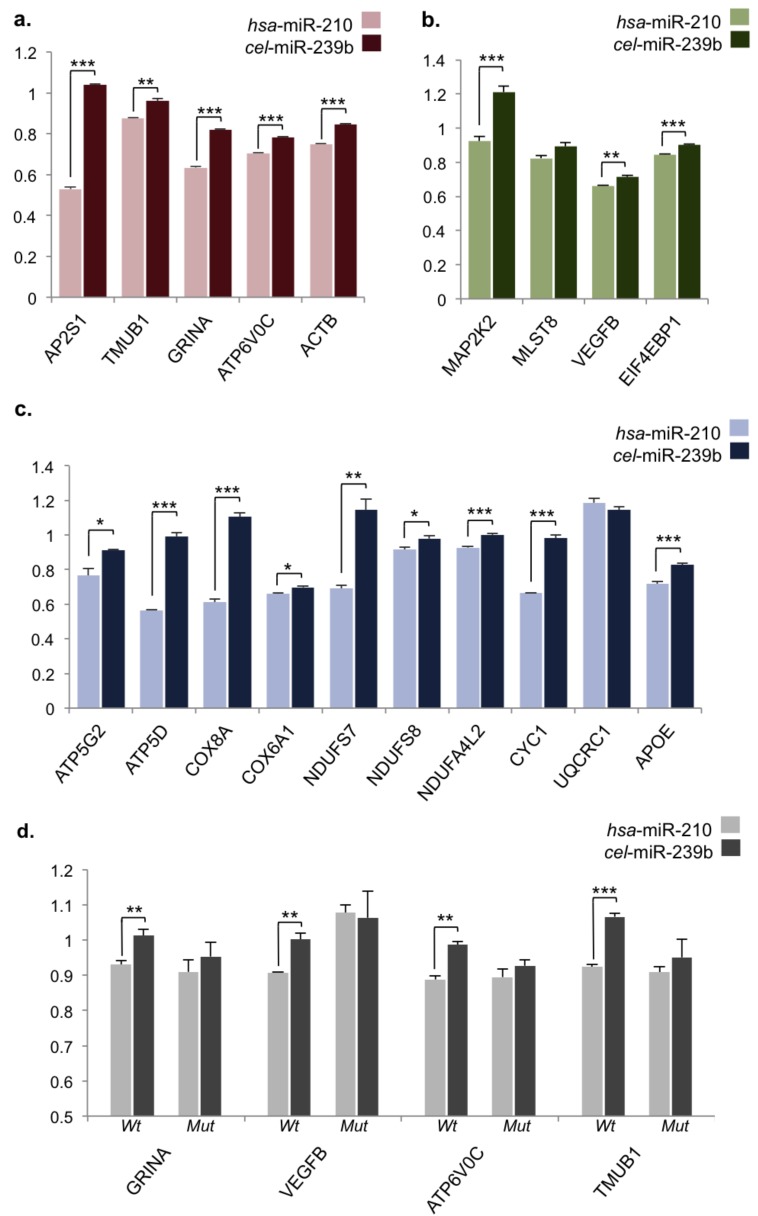
Dual-luciferase assay validation of miR-210 target regulation. (**a**–**d**) Measured *Renilla-firefly* luciferase ratios of target region/ΨCheck2 vector constructs transfected into Cos-7 cells alone or in combination with hsa-miR-210 mimic of cel-miR-239b negative control mimic, normalized to native ΨCheck2 and plasmid-only values. (**a**) 3′ untranslated regions (UTRs) of neuronal/synaptic plasticity genes; (**b**) 3′-UTRs of cancer-related mTOR/VEGF signaling pathway genes; (**c**) 3′UTRs of metabolic pathway genes; (**d**) Short microRNA recognition elements (MREs) in predicted target gene 3′UTRs (*Wt*) and mutated MRE sites (*Mut*). *Y*-axis = *Renilla-firefly* luciferase ratio in all graphs; error bars represent standard error; * *p* < 0.05, ** *p* < 0.01, *** *p* < 0.001; *n* = 4 biological replicates, two sample *t*-test.

As miRNAs are known to primarily target genes through 3′-UTRs, primers flanking these regions were designed for 19 selected genes of interest and whole 3′-UTRs were amplified and cloned into the ΨCheck2 vector backbone. To assay luciferase activity, the Cos-7 cell line, derived from African green monkey kidneys, was chosen as a readily transfected cell system that possesses mammalian RISC components required for assessing miRNA functionality. The primate miRNA genome also displays significant diversification from the human, so utilizing a primate cell line may potentially reduce background noise from endogenous regulatory factors [48]. To assay gene regulation, constructs were transfected into the mammalian Cos-7 cell line either alone or co-transfected with hsa-miR-210 mimic or cel-miR-239b negative control mimic. Luciferase expression was measured after 24 h and all data were normalized to native ΨCheck2 vector samples to account for effects of mimic transfection and potential interactions with foreign sequences in the ΨCheck2 vector before normalizing data for individual constructs to plasmid-only transfected samples. From this we found that the majority of genes we tested (17/19, >89%) were significantly downregulated by miR-210 through their respective 3′-UTRs (Figure 2). Significantly regulated genes associated with neuronal/synaptic plasticity included those mentioned previously among the top enriched target genes from pull-down data (*GRINA*, *AP2S1*, and *TMUB1*) as well as the OXPHOS-related *ATP6V0C*, a subunit of vacuolar ATPase (v-ATPase), involved in synaptic vesicle function, and the actin subunit *ACTB*, whose homolog was also identified as a potential target of miR-210 in the honeybee (Table 1, Figure 2a) [6,49]. MAPK/VEGF signaling-related genes *EIF4EBP1*, *VEGFB*, and *MAP2K2* as well as numerous neurodegenerative-related genes were also validated as significantly regulated by miR-210, including OXPHOS genes (*ATP5G2*, *ATP5D*, *COX8A*, *COX6A1*, *NDUFS7*, *NDUFS8*, *NDUFA4L2*, and *CYC1*) and the apolipoprotein E gene, *APOE*, the major genetic risk factor for AD (Figure 2b,c) [50]. To confirm the specificity of luciferase assays, we also looked for predicted miR-210 recognition elements (MREs) in 3′-UTRs of significantly downregulated genes of interest. We identified four MREs using the hybridization-based algorithm RNAHybrid in 3′-UTRs of *GRINA*, *VEGFB*, *ATP6V0C*, and *TMUB1* (Figure S4). MRE constructs were assayed alongside mutant MREs with G/C>A point mutations introduced in binding regions, and we confirmed that downregulation by miR-210 through these MREs was disrupted by mutating these sites (Figure 2d,e). This suggests that the majority of gene targets identified through our biotin pull-down approach are genuine regulatory targets and that miR-210 may regulate a number of key genes involved with neuronal function and neurodegeneration.

## 3. Discussion

Here we generated a global targeting picture of miR-210 in a human neuronal system and identified overall functional and disease pathways that are potentially regulated by miR-210 through this targeting network. Among already known roles of miR-210 and diseases associated with its overexpression, a number of novel miR-210 regulatory pathways have been identified, including neurodegenerative disorders. Additionally, we have also validated that miR-210 is able to directly regulate various genes associated with metabolic function and MAPK/VEGF signaling as well as a number of key synaptic genes through their 3′-UTRs. 

Although miR-210 has been studied across numerous cell and tissue types under hypoxic conditions, little is known about its potential role in neuronal plasticity under physiological conditions. Given that the brain is the largest source of oxygen consumption in the body and different brain regions display wide variation in oxygen tension under physiological conditions (0–99 mm Hg), we expect that miR-210, which is known to regulate oxidative metabolism and is itself regulated by oxygen, may have a significant role in neuronal function [51]. Our study supports this hypothesis, extending in vivo evidence of miR-210 dysregulation in neuronal disorders and during learning and memory. Previously we showed that miR-210 was upregulated following long-term memory formation in the honeybee using an olfactory conditioning paradigm, and, conversely, deficits in learning and memory were observed when miR-210 was knocked down in honeybees using an antagomir approach [6]. In a separate study, it was also shown that miR-210 upregulation is associated with age-related behavioral changes in honeybees, being upregulated in aged bees following their transition from nursing to foraging behavior [52]. In addition, upstream of miR-210, HIF-1α has also been found to be upregulated by various forms of neuronal activity in vivo, including induction of seizures in rats, in mice subject to environmental enrichment, and following long-term memory formation in the Morris water maze [53,54,55].

Throughout the brain, energy metabolism is highly dynamic, driven by increased neuronal activation, which increases metabolic demands through adenosine triphosphate (ATP) dependent processes such as maintenance of electrochemical gradients, protein synthesis, and axoplasmic transport [56]. To meet varying metabolic demands, neurovascular coupling has evolved to increase blood flow and blood volume to areas of increased local activity [57]. Despite these compensatory mechanisms, there is increasing evidence that oxidative metabolism remains elevated following blood flow and blood volume returning to baseline levels, and it seems likely that with continued neuronal activation, oxygen levels will be depleted, potentially having secondary effects on hypoxia-inducible gene expression [58].

From our gene ontology analysis of pull-down miR-210 target genes, one of the major functionalities we identified was oxidative phosphorylation, which is consistent with some of the known roles of miR-210 in other cell types, and target genes annotated to enriched neurodegenerative KEGG pathways also overlapped significantly with OXPHOS genes [8]. Metabolic decline and oxidative stress are hallmarks of neurodegeneration, and neurodegenerative diseases are strongly associated with degeneration or dysfunction of the brain microvasculature, which diminishes cerebral blood flow and oxygen supply. While hypoxia-regulated genes such as miR-210 may have a role in regulating metabolism in response to hypoxic stress across various tissues, we expect this will be especially critical within the brain not just during oxidative stress, but during normal brain function. Just as energy metabolism and demand are dynamic throughout the brain, metabolic activity is heterogeneous throughout a single neuron, with metabolic machinery distributed locally within axons and dendrites [59]. Regulatory mechanisms of neuronal metabolism therefore need to be activity-inducible and able to be locally switched on/off. We know already that local regulation of miRNAs occurs in response to activity in neurons and that miR-210, as the master miRNA regulator of hypoxia, is a strong candidate as a regulatory molecule that may be involved in directing neuronal plasticity by directing metabolic resources to active synapses.

Another significant pathway identified from ontology analysis was mRNA translation, which is a function not previously associated with miR-210 but one that is critical to plasticity. Regulation of translation is also linked to metabolism and hypoxic stress, being an energy-expensive process that is downregulated during hypoxia [60]. In neurons, like oxidative metabolism, translation also occurs locally within synapses and dendrites and in an activity-dependent manner. Numerous mechanisms of translational control are known to be involved in activity-dependent neuronal plasticity, and miRNAs are important regulators of translational proteins, as their synthesis does not require translational machinery. One important mechanism regulating the translation of gene expression during learning and memory is the inhibition of cap-dependent translation initiation by eIF4E, regulated by a family of binding proteins (4E-BPs). The best characterized of these is EIF4EBP1 (4E-BP1), which binds with high affinity to inhibit eIF4E in its hypophosphorylated form and with low affinity following activity-induced hyperphosphorylation, promoting an increase in translation [25].

We have shown that miR-210 directly interacts with and downregulates *4E-BP1* through its 3′-UTR along with a number of other genes of interest to synaptic function, including *GRINA*, *TMUB1*, and *AP2S1*. *GRINA* is a subunit of the NMDA receptor complex, which is a critical target of regulation during synaptic plasticity and has been associated with epilepsy and dysregulation by long-term consolidation in fear conditioning [28,61,62]. Both *TMUB1* and *AP2S1* are synaptosomal transmembrane receptor-binding proteins involved with regulating trafficking and surface expression of AMPA and GABA_A_ receptors [26,27,63]. Also validated as a target gene of miR-210 was *ATP6V0C*, one of three subunits of the v-ATPase identified from pull-down data. In neurons v-ATPase is involved in synaptic function required for loading of neurotransmitter into synaptic vesicles, and potentially involved in vesicle fusion [49]. Other genes of interest validated as miR-210 targets here were *ACTB* and *APOE*. *ACTB* regulation is a critical aspect of plasticity, and has been consistently shown to be regulated during learning and memory and various forms of plasticity [28,64,65]. We also previously identified actin as a potential regulatory target of miR-210 in the honeybee in the homologous *Act5C* gene and observed inversely correlated expression of miR-210 and *Act5C* following olfactory conditioning [6]. *APOE* is a major genetic predictor of AD, having both protective and risk factor allelic variants and effecting amyloid β plaque clearance as well as glutamate receptor function in neurons [66]. Based on the identification of a number of novel miR-210 targets involved with plasticity and/or synaptic function, this may indicate novel functional effects of miR-210 in neuronal systems that may be further elucidated by cellular functional characterization as well as more extensive behavioral profiling in mammalian models.

The same gene regulatory dynamics discussed here in relation to plasticity will also be relevant to neurodegenerative disorders as well as ischemic injury and other forms of neurological traumas that result in hypoxic damage. As we anticipate that miR-210 may promote plasticity changes through local metabolic regulation during normal brain function, we similarly expect that during periods of severe hypoxia or oxidative stress, when miR-210 expression is more severely dysregulated, altered regulation of downstream miR-210 genes may contribute to cognitive deficits associated with neurological damage and disease. This is supported by the enrichment of neurodegenerative diseases among miR-210 targets and metabolic decline being a major pathological component of neurodegeneration [67,68]. Despite substantial research on the pathology of neurodegenerative diseases, there are still major gaps in our understanding of their underlying causes and neuronal activity-dependent “use it or lose it” nature. Identifying the molecular links between neuronal activation, altered metabolism, and plasticity changes is therefore critical to understanding how dysregulated metabolism may lead to cognitive deficits in neurodegenerative conditions. Characterizing genes such as miR-210 and other sensors of metabolism may provide significant biological insight into the mechanisms involved in translating environmental signal into neural response and, consequently, cognitive function.

In vivo, miR-210 knockout mouse models have been created that are both viable and fertile, with no gross abnormalities, indicating that it does not play an essential developmental role [69,70]. Functional assessment of adult miR-210 knockout mice, however, did identify increased resistance to pulmonary hypertension and hypoxia-induced metabolic dysfunction in lung tissue [71]. A second study discovered an age-associated autoimmunity phenotype in these mice, with aged miR-210 knockout mice developing spontaneous autoantibodies and possessing increased germinal centre B-cells [72]. Enrichment of oxidative metabolism processes identified here from pull-down analyses is consistent with some of the functional effects of miR-210 knockout observed in vivo. The identification of a phenotype in aged mice could also be indicative of disruptions to other miR-210 regulatory pathways manifesting with age, which may be relevant to neurodegenerative diseases. However, further behavioural characterization of adult and aged miR-210 knockout mice would be required to identify potential effects of miR-210 in brain plasticity and age-related cognitive decline.

## 4. Materials and Methods 

Unless otherwise stated, cell culture media and RNA reagents were purchased from Thermo-Fischer Scientific (Melbourne, Australia). All chemicals were purchased from Sigma-Aldrich (Sydney, Australia), cloning reagents and restriction enzymes were from New England Biolabs (NEB, Ipswich, MA, USA), and antibodies were sourced from Abcam (Cambridge, MA, USA).

### 4.1. Biotin Pull-Down Assay

SH-SY5Y cells purchased from Sigma-Aldrich (Sydney, Australia) were transfected in triplicate with 600 pmoles of single-stranded biotinylated–miR-210-3p mimic (bi-miR-210) (GenePharma, Shanghai, China; 5’CUGUGCGUGUGACAGCGGCUGA–biotin, accession: MIMAT0000267) or bi-miR-239b-5p mimic (Genepharma; 5’UUUGUACUACACAAAAGUACUG–biotin, accession: MIMAT00 00295). Then 150 µL of Dynabeads M-280 streptavidin was prepared for RNA manipulation according to the manufacturer’s directions. Beads were blocked overnight on rotation at 4 °C with 1 mg/mL yeast transfer RNA (tRNA) and 1 mg/mL bovine serum albumin (BSA). After 24 h, cells were trypsinized, spun down, and washed twice in ice-cold phosphate-buffered saline (PBS) before being lysed on ice for 30 min in cell lysis buffer containing 10 mM Tris-HCl (pH 7.5), 10 mM KCl, 1.5 mM MgCl_2_, 5 mM dithiothreitol (DTT), 0.5% IGEPAL CA-630, 50 U/mL RNase Out, and 1× complete mini-protease inhibitor cocktail (Roche, Sydney, Australia). Blocked beads were washed and resuspended in 450 µL of wash buffer (lysis buffer with NaCl adjusted to 1 M). Lysates were cleared at 5000× *g* for 5 min at 4 °C, and supernatant was removed and adjusted to 1 M NaCl/450 µL. Cell lysate was incubated with beads on rotation for 30 min at room temperature and subsequently washed 3 times in wash buffer before being resuspended in 250 µL of RNAse/DNase free water.

### 4.2. RNA Extraction, Complementary DNA Synthesis, and qPCR

RNA extraction was performed using 1 mL of TRIzol reagent or 750 µL for biotin pull-down samples. RNA extraction was performed according to the manufacturer’s instructions, with all steps carried out on ice and RNA precipitated overnight at −20 °C with 20 µg of glycogen. Samples were then quantified on a NanoDrop Lite spectrophotometer (Thermo-Fischer Scientific, Melbourne, Australia) and Qubit fluorometer (Thermo-Fischer Scientific, Melbourne, Australia) using the high-sensitivity RNA assay. Synthesis of complementary DNA (cDNA) for protein-coding gene expression was performed using SuperScript III reverse transcriptase (RT) according to the manufacturer’s directions, using oligo dT (total RNA) or random primers (pull-down mRNA). Polymerase chain reactions (PCRs) were performed on the Roche LightCycler 480 system (Roche, Sydney, Australia) using the LightCycler 480 SYBR Green I Master (Roche, Sydney, Australia) with gene-specific primers (Table S7).

### 4.3. RNA Sequencing

RNA samples were run on an Agilent 2100 Bioanalyzer (Santa Clara, CA, USA) to determine RNA quality, and samples were enriched for intact mRNA using poly(A) beads to minimize ribosomal RNA contamination. A total of 10 ng of RNA was used for cDNA synthesis using the TruSeq stranded mRNA Library Prep Kit (Illumina, San Diego, CA, USA), and paired-end sequencing reads were generated on an Illumina HiSeq 2000 (Illumina, San Diego, CA, USA). RNA sequencing results were trimmed of all adapter sequences and low-quality bases using Trimmomatic (v0.33) [73], and any sequences < 36 base pairs (bp) were discarded. Sequences were aligned with the TopHat (v2.1.0) and Bowtie2 (v2.2.6) index tools [74] using the UCSC hg19 reference and annotation and alignment files sorted using SAMtools (v1.2) [75]. Count files were generated using HTSeq [76] and analyzed using the DESeq2 package (v.1.10.11) [77]. The list of miR-210 significantly enriched genes was obtained by a “greater” threshold-based Wald test of significance on DESeq2 results with an log fold change (lfc) threshold of 1, using an adjusted *p*-value of <0.05 and a log2 fold change >1.5 as cutoffs for significance.

### 4.4. Ontology Analysis

To develop a list of genes differentially regulated during long-term consolidation in vivo, relevant studies were found through PubMed searches and included on the basis of transcriptome/proteome expression analysis following various forms of consolidation (Microarray/RNASeq/2DGE) and availability of data. Gene/array IDs were converted to homologous human IDs using Ensembl BioMart (release 81). Gene ontology for enriched biological processes and KEGG pathways were generated using the DAVID functional annotation tool with default settings and significance cutoffs (*p*-value < 0.1). Transcription factor binding site (TFBS) enrichment was determined using the oPOSSUM single-site analysis online tool; parameters were set to detect TFBSs within 5000 bp of upstream sequence and 2000 bp of downstream sequence, and cutoffs for significant enrichment were Z-score > 15 and target TFBS nucleotide rate/background TFBS nucleotide rate > 1.5.

### 4.5. Cloning

Primers flanking the 3′UTRs of selected target genes were designed with 5′ Xho I and 3′ Not I restriction sites flanking forward and reverse primers (Table S8). For MREs/short 3′UTRs, the entire sequence with 5′ Xho I and 3′ Not I complimentary overhangs was designed as single-stranded synthetic oligos (Table S9). In a thermocycler, oligos were phosphorylated using T4 PNK in T4 ligase buffer and annealed (37 °C for 30 min, 95 °C for 5 min, ramp to 25 °C/1 °C min^−1^). Q5 high-fidelity DNA polymerase was used to amplify 3′UTRs and gene fragments according to the manufacturer’s directions. Purified PCR products and ΨCheck2 vector (Promega, Madison, WI, USA) were double-digested with Xho I and Not I-high fidelity (Not I-HF) restriction enzymes in Cut-Smart buffer, and digested ΨCheck2 was dephosphorylated using rAPid Alkaline Phosphatase (Roche, Sydney, Australia). Digested products and synthetic oligos were ligated into the ΨCheck2 vector using T4 DNA ligase. Ligated constructs were transfected into chemically competent *Escherichia coli* and inserts verified by Sanger sequencing.

### 4.6. Cell Culture and Transfection

SH-SY5Y cells were maintained in 5% CO_2_ at 37 °C in DMEM/F-12/15 mM HEPES supplemented with 10% fetal bovine serum (FBS) and 100 U/mL Pen-Strep. SH-Y5Y cells were transfected using Lipofectamine 2000 (Thermo-Fischer Scientific, Melbourne, Australia) at a final concentration of 2.5 µL/mL according to the manufacturer’s directions. Cos-7 cells were maintained in 5% CO_2_ at 37 °C in DMEM/10% FBS/100 U/mL Pen-Strep and transfected according to the manufacturer’s directions using Lipofectamine LTX with Plus reagent (Thermo-Fischer Scientific, Melbourne, Australia) at a final concentration of 4 µL/mL and 1 µL/mL, respectively.

### 4.7. Dual Luciferase Assays

Cos-7 cells were plated at 1 × 10^4^ cells/well in 48-well plates in media without antibiotics. Cells were attached overnight and transfected with 200 ng of plasmid with/without 60 pmoles of miR-210 mimic or miR-239b mimic before being incubated for 24 h. Cell lysates were prepared using the Dual Luciferase Reporter Assay System (Promega, Madison, WI, USA) as per the protocol and transferred to a 96-well Lumitrac 200 plate (Sigma-Aldrich, WI, USA). Luciferase activity was measured using a CLARIOstar microplate reader (BMG LABTECH, Ortenberg, Germany). *Renilla* luciferase was normalized to firefly luciferase, and luciferase ratios for constructs were normalized to native ΨCheck2 values.

### 4.8. Statistics

All statistical analyses were carried out in R-studio at a 95% confidence interval (α = 0.05). Homogeneity of variance and data normality distribution was determined using Levene and Shapiro-Wilk tests, respectively. Specific means tests and appropriate post hoc analyses were performed as indicated in the text.

## Figures and Tables

**Table 1:** Top miR-210 targets identified from pull-down RNA sequencing analysis. Top 20 genes most significantly enriched from biotin–miR-210 pull-down. **FC** = fold change, padj = adjusted *p*-value. Rows highlighted in gray indicate genes with a known role in plasticity, **bold** indicates genes previously identified as differentially regulated by long-term consolidation, ▲ indicates genes validated by real-time quantitative polymerase chain reaction (qPCR).

Gene Name	Gene ID	padj	FC
Oligosaccharyltransferase complex subunit 4 (non-catalytic) ▲	*OST4*	1.96 × 10^−91^	7.31
Ribosomal protein L28 ▲	*RPL28*	1.08 × 10^−54^	6.02
6-Phosphogluconolactonase ▲	*PGLS*	1.74 × 10^−42^	6.27
**NME/NM23 Nucleoside diphosphate kinase 4**	***NME4***	**3.36 × 10^−30^**	**4.97**
**NADH: Ubiquinone oxidoreductase core subunit S7 ▲**	***NDUFS7***	**9.47 × 10^−29^**	**5.07**
mTOR Associated protein, LST8 homolog ▲	*MLST8*	3.47 × 10^−27^	4.41
Pancreatic progenitor cell differentiation and proliferation factor	*PPDPF*	2.53 × 10^−26^	4.29
Solute carrier family 39 (Zinc transporter), member 4	*SLC39A4*	7.50 × 10^−26^	5.42
**Glutamate receptor, ionotropic, *N*-methyl d-aspartate-associated protein 1 (Glutamate binding) ▲**	***GRINA***	**1.02 × 10^−25^**	**4.16**
Adaptor related protein complex 2 Sigma 1 subunit	*AP2S1*	1.46 × 10^−25^	4.50
**Phosphatidylethanolamine *N*-methyltransferase**	***PEMT***	**1.46 × 10^−25^**	**4.80**
Protein phosphatase 1, catalytic subunit, alpha isozyme	*PPP1CA*	9.00 × 10^−25^	4.81
Vascular endothelial growth factor B	*VEGFB*	1.25 × 10^−24^	4.40
SH3 Domain binding glutamate-rich protein like 3 ▲	*SH3BGRL3*	6.90 × 10^−24^	3.98
Transmembrane and ubiquitin-like domain containing 1	*TMUB1*	3.78 × 10^−23^	4.25
**Guanine nucleotide binding protein (G protein), gamma 5**	***GNG5***	**4.70 × 10^−23^**	**5.21**
CD320 Molecule	*CD320*	4.72 × 10^−23^	4.72
Eukaryotic translation initiation factor 4E binding protein 1 ▲	*EIF4EBP1*	5.55 × 10^−23^	4.04
Ganglioside induced differentiation associated protein 1-like 1	*GDAP1L1*	3.49 × 10^−22^	4.21
Lymphocyte antigen 6 complex, locus E	*LY6E*	9.13 × 10^−22^	4.54

Abbreviations: NME, non-metastatic cell expressed; NADH, nicotinamide adenine dinucleotide; mTOR, mammalian target of rapamycin.

**Table 2:** Gene ontology terms enriched among miR-210 target genes. Most significantly enriched biological processes and Kyoto Encyclopedia of Genes and Genomes (KEGG) pathways from miR-210 pull-down targets. Generated using the Database for Annotation, Visualization, and Integrated Discovery (DAVID) functional annotation tool. Count indicates number of miR-210 target genes associated with each annotation; significance cutoff = *p*-value < 0.1.

**Biological Process**	**GO Number**	**Count**	***p*-Value**
Translational elongation	GO:0006414	16	1.03 × 10^−6^
Translation	GO:0006412	26	1.03 × 10^−4^
Macromolecular complex assembly	GO:0065003	38	1.31 × 10^−3^
RNA elongation	GO:0006354	8	1.33 × 10^−3^
Protein complex assembly	GO:0006461	31	1.37 × 10^−3^
Protein complex biogenesis	GO:0070271	31	1.37 × 10^−3^
Generation of precursor metabolites and energy	GO:0006091	22	1.68 × 10^−3^
Phospholipid biosynthetic process	GO:0008654	11	1.95 × 10^−3^
Mitochondrion organization	GO:0007005	13	2.03 × 10^−3^
Macromolecular complex subunit organization	GO:0043933	39	2.23 × 10^−3^
**KEGG Pathway**	**KEGG Number**	**Count**	***p*-Value**
Ribosome	hsa03010	15	3.14 × 10^−6^
Huntington’s disease	hsa05016	16	2.76 × 10^−3^
Purine metabolism	hsa00230	14	4.44 × 10^−3^
Pyrimidine metabolism	hsa00240	10	8.61 × 10^−3^
Oxidative phosphorylation	hsa00190	12	8.90 × 10^−3^
Glutathione metabolism	hsa00480	7	1.02 × 10^−2^
Alzheimer’s disease	hsa05010	13	1.82 × 10^−2^
VEGF Signaling pathway	hsa04370	8	2.12 × 10^−2^
mTOR Signaling pathway	hsa04150	6	4.35 × 10^−2^
Renal cell carcinoma	hsa05211	7	4.56 × 10^−2^

Abbreviations: VEGF, Vascular endothelial growth factor; mTOR, mammalian target of rapamycin.

## Supplementary Materials

The following are available online at http://www.mdpi.com/2311-553X/4/2/10/s1: Figure S1. Quantitative real-time PCR validation of pull-down genes; Figure S2. KEGG pathway analysis of transcription factors with enriched binding sites among miR-210 target genes; Figure S3. Targets in oxidative phosphorylation pathway; Figure S4. Predicted miR-210 miRNA recognition elements; Table S1. RNAseq significantly enriched genes; Table S2. Long-term consolidation—gene regulation; Table S3. Gene ontology—biological processes; Table S4. Gene ontology—KEGG pathways; Table S5. Enriched TFBS; Table S6. Target genes contributing to functional KEGG pathways; Table S7. Gene-specific human primers used in qPCR; Table S8. Primer sequences for cloning 3′-UTRs; Table S9: Oligo sequences for cloning short MREs and 3′UTRs.
