# Supplementary material for: Hypoxia-Induced MicroRNA-210 Targets Neurodegenerative Pathways
Source: Noncoding RNA. 2018 Mar 27;4(2):10. doi: 10.3390/ncrna4020010 (PMC6027187; doi:10.3390/ncrna4020010)
Supplement: Supplementary file 1 [file ncrna-04-00010-s001.zip › ncrna-282068-Supplementary.pdf]

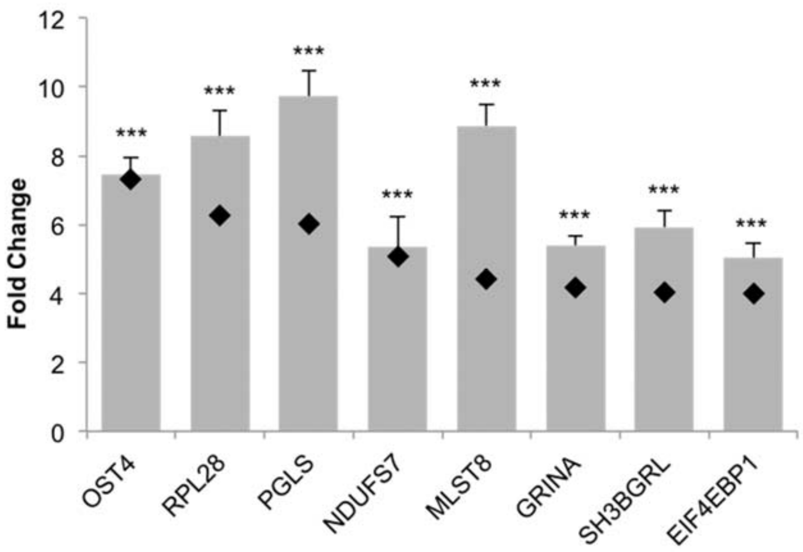

Figure S1. Quantitative real-time PCR validation of genes identified by RNAseq. Pull-down samples were normalized to untransfected control lysate samples and fold change calculated as enrichment of genes in hsa-miR-210 pull-down samples compared to cel-miR-239b samples. Errors represent standard deviation, *** p <0.001, n = 3, Welsh two sample t-test, ◈ indicates corresponding fold change from RNAseq data.

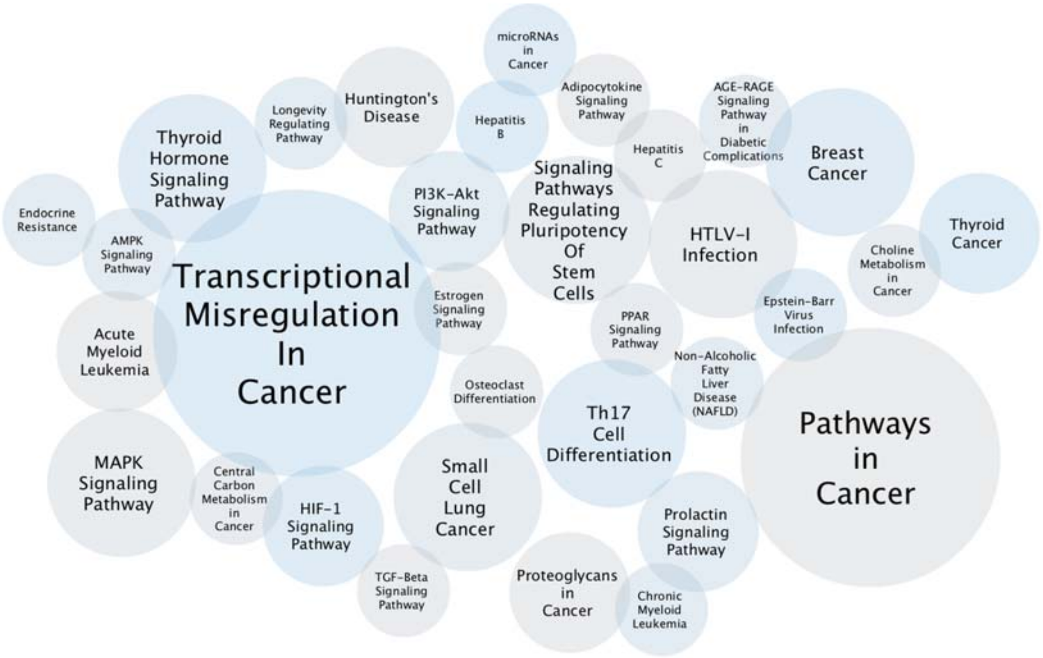

Figure S2. Enriched transcription factor binding site KEGG pathway terms. Graphical representation of KEGG pathway terms annotated to multiple transcription factors with enriched binding sites among miR-210 target genes. Node size correlates to number of associated transcription factors.

Table S6. Target genes contributing to enriched functional KEGG pathways. Potential key target genes were selected based on multiple annotations among KEGG pathways. Metabolic genes overlapping across oxidative phosphorylation and neurodegenerative pathways are highlighted in dark blue, cancer-associated genes overlapping with mTOR and VEGFB signalling pathways are highlighted in dark green. Vacuolar ATPase genes highlighted in dark red are associated with synaptic vesicle recycling.

Huntington's disease	Oxidative phos.	Alzheimer's disease	VEGF signalling pathway	mTOR signalling pathway	Renal cell carcinoma	Parkinson's disease	Acute myeloid leukaemia	Bladder cancer
AP2S1	ATP5D	APOE	BAD	EIF4EBP1	HRAS	ATP5D	BAD	HRAS
ATP5D	ATP5G2	ATP5D	HRAS	MAPK3	MAP2K2	ATP5G2	EIF4EBP1	MAP2K2
ATP5G2	ATP6V0C	ATP5G2	HSPB1	MLST8	MAPK3	COX6A1	HRAS	MAPK3
CLTB	ATP6V0E2	BAD	MAP2K2	PGF	PGF	COX8A	MAP2K2	PGF
COX6A1	ATP6V1F	COX6A1	MAPK3	RPS6KB2	TCEB2	CYC1	MAPK3	VEGFB
COX8A	COX6A1	COX8A	MAPKAPK3	VEGFB	TGFB1	NDUFA4L2	RPS6KB2	
CYC1	COX8A	CYC1	RAC3		VEGFB	NDUFS7		
GPX1	CYC1	GAPDH	SPHK2			NDUFS8		
NDUFA4L2	NDUFA4L2	MAPK3				UBE2L6		
NDUFS7	NDUFS7	NDUFA4L2				UQCRC1		
NDUFS8	NDUFS8	NDUFS7						
POLR2E	UQCRC1	NDUFS8						
POLR2I		UQCRC1						
POLR2J								
POLR2L								
UQCRC1								

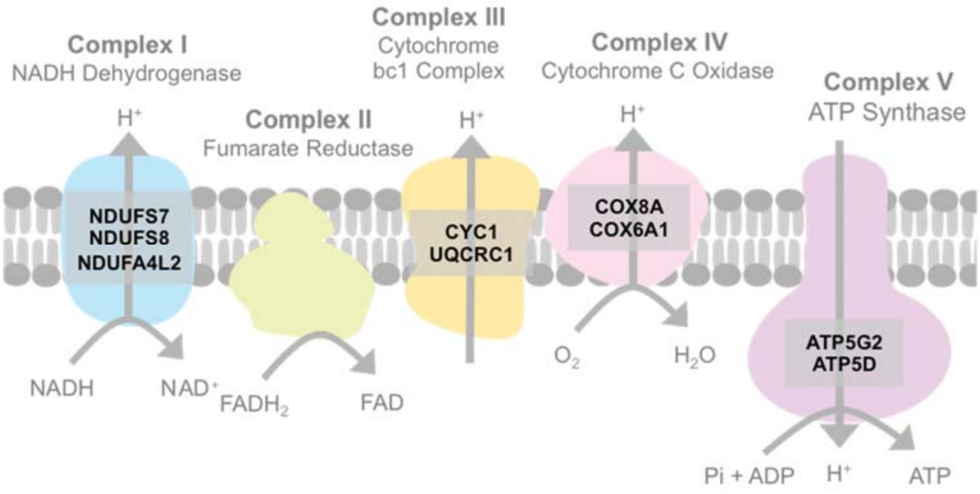

Figure S3. Targets in oxidative phosphorylation pathway. A number of identified miR-210 pull-down targets are subunits of large protein complexes that make up the OXPHOS electron transport chain. Multiple subunits of complexes I, III, IV, and V were significantly enriched by miR-210, all are nuclear encoded OXPHOS genes.

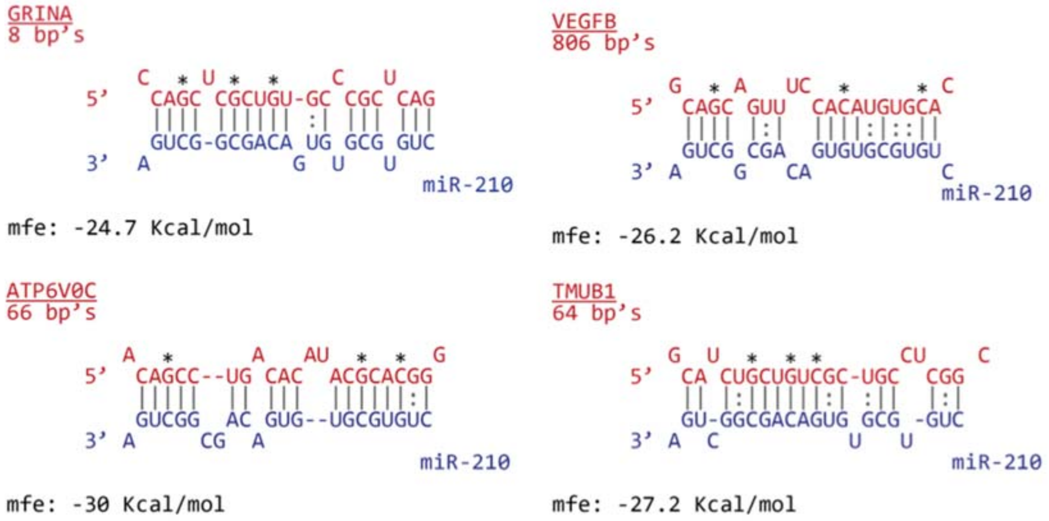

Figure S4. Predicted miR-210 miRNA recognition elements (MREs) in selected targets. MRE predictions generated using the RNAhybrid algorithm. Specified **bp's** indicates position downstream from stop codon, **'|'** indicates complimentary base-pairing, **':'** indicates G:U wobble base-pairing, **mfe** = minimum free energy, '*****' indicates position of introduced point mutations; G/C>A.

Table S7. Gene-specific human primers used in q-PCR.

Gene	***Sequence 5'>3'***
OST4	F: TCGCCATCTTCGCCAACAT
	R: GACGGCCACGTAGTGATAGAG
RPL28	F: CTTCCGCTACAACGGACTGAT
	R: ATGACCACCACGACACCTTTG
PGLS	F: CATCCCGGTTTTCGACCTG
	R: TCGGGGAGTCACTGATGGG
NDUFS7	F: CTTCGCAAGGTCTACGACCAG
	R: GGAATAGTGGTAGTAGCCTCCTC
MLST8	F: GGGACTTGAAAACAGACCACA
	R: CCGTCAGATTCCAGACATAGCA
GRINA	F: ATGATCGCCAGCTTCTACAAC
	R: GTGAAGTCGTAGCGGGTCTG
SH3BGRL3	F: CTCCCGCGAAATCAAGTCCC
	R: CCCGTTGACAATCTGGGGTG
EIF4EBP1	F: CTATGACCGGAAATTCCTGATGG
	R: CCCGCTTATCTTCTGGGCTA

Table S8. Primer sequences for cloning 3'UTRs. Red text indicates restriction enzyme sites, gray text indicates restriction site flanking regions, and sequences in black are gene specific regions.

Target	***Primer: Sequence 5' > 3'***
	Sequencing
ΨCheck2	F: GGACGCTCCAGATGAAATG<br>R: CAAACCCTAACCACCGCTTA
	PCR Amplification
AP2S1<br>*3'UTR*	F: CAGTCAAGCTCGAGCAGGCTGATGCTACAGTCCCTG<br>R: TCAACTGCGGCCGCGTCGCAGGACCACAGGTTTATTG
TMUB1<br>*3'UTR*	F: CAGTATTGCTCGAGCAGATGTACCGCCCGTAGTGC<br>R: ATCATTTAGCGGCCGCATCACTTCACAGTTACTTTAATCTGCAC[illegible]
GRINA<br>*3'UTR*	F: CAGTATCACTCGAGCAGAGGAGTAGCCGAGCTCCAG<br>R: ATAGTTTAGCGGCCGCGTCTGCAGAGAGCAAATCCCATTTATTG
ATP6V0C<br>*3'UTR*	F: CAGTGACTCTCGAGCAGAAGTAGACCCTCTCCGAGC<br>R: TCACTGGCGGCCGCGTAGCCCGTCACATCCAAGAAC
ACTB<br>*3'UTR*	F: CAGTATCACTCGAGCAGGGCGGACTATGACTTAGTTG<br>R: ATCATTTAGCGGCCGCGTCGGTGTGCACTTTTATTCAACTG
MLST8<br>*3'UTR*	F: GAATGACTCTCGAGCAGCTGCCTGGCCTTCAATGAC<br>R: TCAACTGCGGCCGCGTCCCGAGCAACATCTGCGTG
VEGFB<br>*3'UTR*	F: CAGTCATACTCGAGCAGGAGCTCAACCCAGACACC<br>R: ATCATTAGCGGCCGCGTCCCAGAGAAGTTTGAGACTATCTTTAC
EIF4EBP1<br>*3'UTR*	F: CAGTGACTCTCGAGCAGAGCACCAGCCATCGTGTG<br>R: TCACTGGCGGCCGCGTCCTTGGCCCTAGGGCGAAG
MAP2K2<br>*3'UTR*	F: CAGTGACTCTCGAGCAGGTGACAGTGGCCGGGCTC<br>R: TCACTGGCGGCCGCGTAGCCTCTGAGACCACACACAGCA
ATP5G2<br>*3'UTR*	F: CAGTGACTCTCGAGCAGATGTGAAGGAGCCGTCTC<br>R: TCACTGGCGGCCGCGTATCTCTGTCAAACCCTGAGC
ATP5D<br>*3'UTR*	F: CAGTGACTCTCGAGCAGGAGTAGGCGGTGCGTACC<br>R: TCACTGGCGGCCGCGTACAGGCTTCCGGGTCTTTAATGG
COX8A<br>*3'UTR*	F: CAGTGACTCTCGAGCAGACAGGAGGCCAGAGTGAAG<br>R: TCACTGGCGGCCGCGTACCACCAAGCAGGGTCAGT
COX6A1<br>*3'UTR*	F: GAATGATCTCGAGCAGAGAGAATCTGGACCACTACC<br>R: TCACACGCGGCCGCGTCTATTTAAGCCATCTCCTGCCA
NDUFS7<br>*3'UTR*	F: CAGTCAAGCTCGAGCAGGATCTGGTACCGCAGGTAG<br>R: TCAACTGCGGCCGCGTCGAGGGCAGGTTTATTGACAAC
NDUFA4L2<br>*3'UTR*	F: CAGTGACTCTCGAGCAGCAGACTTCTAAGCCAGGCTGG<br>R: TCACTGGCGGCCGCGTCGTATTTTGCCCACGGCTGC
CYC1<br>*3'UTR*	F: CAGTGACTCTCGAGCAGCCTGTCCAGTGTCTGCTTGC<br>R: TCACTGGCGGCCGCGTACCATGATGGGGGCTGAAGG
UQCRC1<br>*3'UTR*	F: CAGTGACTCTCGAGCAGGGGAAGCCTATGTAAGCAAG<br>R: TCACTGGCGGCCGCGTATCACTTCTCAGCAGAGGATT
APOE<br>*3'UTR*	F: GAATGACTCTCGAGCAGAATCACTGAACGCCGAAGC<br>R: CATGCAGCGGCCGCGTATGATGCGTGAAACTTGGTGAATC

Table S9. Oligo sequences for cloning short MREs and 3'UTRs. Red text indicates restriction enzyme sites, gray text indicates restriction site flanking regions, and sequences in black are annealing regions.

Target	***Oligo: Sequence 5' > 3'***
	Oligo Annealing
NDUFS8 *3'UTR -A*	Top: TCGAGCAGCGCCCCACCGGCCCGCAGCCCCTG<br>Bottom: TTGGGCAGCAGGGGCTGCGGGCCGGTGGGGCGCTGC
NDUFS8 *3'UTR -B*	Top: CTGCCCAATAAAACCACTCCGACCCCACGGAGACGC<br>Bottom: GGCCGCGTCTCCGTGGGGTCGGAGTGGTTTTA
GRINA *MRE*	Top: TCGAGCAGCCAGCTCGCTGTGCCCGCTCAGGACGC<br>Bottom: GGCCGCGTCCTGAGCGGGCACAGCGAGCTGGCTGC
VEGFB *MRE*	Top: TCGAGCAGGCAGCAGTTTCCACATGTGCACGACGC<br>Bottom: GGCCGCGTCGTGCACATGTGGAAACTGCTGCCTGC
ATP6V0C *MRE*	Top: TCGAGCAGACAGCCTGACACATACGCACGGGGACGC<br>Bottom: GGCCGCGTCCCCGTGCGTATGTGTCAGGCTGTCTGC
TMUB1 *MRE*	Top: TCGAGCAGGCATCTGCTGTCGCTGCCTCGGCGACGC<br>Bottom: GGCCGCGTCGCCGAGGCAGCGACAGCAGATGCCTGC
GRINA *Mutant*	Top: TCGAGCAGCCAACTCACTATGCCCGCTCAGGACGC<br>Bottom: GGCCGCGTCCTGAGCGGGCATAGTGAGTTGGCTGC
VEGFB *Mutant*	Top: TCGAGCAGGCAACAGTTTCCAAATGTGAACGACGC<br>Bottom: GGCCGCGTCGTTCACATTTGGAAACTGTTGCCTGC
ATP6V0C *Mutant*	Top: TCGAGCAGACAACCTGACACATACACAAGGGGACGC<br>Bottom: GGCCGCGTCCCCTTGTGTATGTGTCAGGTTGTCTGC
TMUB1 *Mutant*	Top: TCGAGCAGGCATCTACTATAGCTGCCTCGGCGACGC<br>Bottom: GGCCGCGTCGCCGAGGCAGCTATAGTAGATGCCTGC
